# MRI-detected osteitis is not associated with the presence or level of ACPA alone, but with the combined presence of ACPA and RF

**DOI:** 10.1186/s13075-016-1076-0

**Published:** 2016-08-02

**Authors:** Debbie M. Boeters, Wouter P. Nieuwenhuis, Marije K. Verheul, Elize C. Newsum, Monique Reijnierse, René E. M. Toes, Leendert A. Trouw, Annette H. M. van der Helm-van Mil

**Affiliations:** 1 Department of Rheumatology C1-R, Leiden University Medical Center, PO Box 9600, Leiden, 2300 RC The Netherlands; 2 Department of Radiology, Leiden University Medical Center, Leiden, The Netherlands

**Keywords:** Rheumatoid arthritis, ACPA, RF, MRI, Bone marrow edema

## Abstract

**Background:**

In rheumatoid arthritis (RA) bone marrow edema (BME, osteitis) and anti-citrullinated protein antibodies (ACPA) are associated with radiographic progression. ACPA have been associated with BME, but it is unknown if this association is confined to ACPA and BME. We performed cross-sectional analysis of the association of ACPA, rheumatoid factor (RF) and anti-carbamylated protein (anti-CarP) antibodies with BME and other types of inflammation (synovitis, tenosynovitis) detected by magnetic resonance imaging (MRI).

**Methods:**

Disease-modifying antirheumatic drug (DMARD)-naïve patients with early arthritis (n = 589), included in the Leiden Early Arthritis Clinic cohort, underwent contrast-enhanced 1.5 T MRI of unilateral wrist, metacarpophalangeal and metatarsophalangeal-joints at baseline. BME, synovitis and tenosynovitis were scored by two readers. ACPA, rheumatoid factor (RF) and anti-CarP were determined at baseline.

**Results:**

In univariable analyses ACPA-positive patients had higher BME scores than ACPA-negative patients (median 4.5 vs. 2.0, *p* < 0.001), but not more synovitis and tenosynovitis. Also RF (median 3.75 vs. 2.0, *p* < 0.001) and anti-CarP antibodies (median 3.5 vs. 2.5, *p* = 0.012) were associated with higher BME scores. Because the autoantibodies were concomitantly present, analyses were stratified for the presence of different autoantibody combinations. ACPA-positive (ACPA+), RF-negative (RF-), anti-CarP-negative (anti-CarP-) patients did not have higher BME-scores than ACPA-negative (ACPA-), RF-, anti-CarP- patients. However ACPA+, RF-positive (RF+), anti-CarP- patients and ACPA+, RF+, anti-CarP-positive (anti-CarP+) patients had higher BME scores than ACPA-, RF-, anti-CarP- patients (median 5.0 and 4.5 vs. 2.0, *p* < 0.001 and *p* < 0.001). ACPA levels were not associated with BME scores. Analyses within RA- and UA-patients revealed similar results.

**Conclusions:**

The presence of ACPA alone or ACPA level was not statistically significantly associated with BME scores, but the combined presence of ACPA and RF was associated with more BME. This suggests an additive role of RF to ACPA in mediating osteitis.

**Electronic supplementary material:**

The online version of this article (doi:10.1186/s13075-016-1076-0) contains supplementary material, which is available to authorized users.

## Background

Rheumatoid arthritis (RA) is characterized by chronic inflammation of the joints that may result in progressive structural damage. Magnetic resonance imaging (MRI) detects inflammation sensitively [1]. Whereas synovitis and tenosynovitis can also be evaluated by other imaging modalities, such as ultrasound, MRI is the only modality that depicts bone marrow edema (BME). Histopathology studies in RA have shown that BME lesions consist of infiltration by leucocytes and an increased number of osteoclasts [2–4]. Therefore, BME has also been named osteitis. These data suggest a link between BME and structural damage in RA. Indeed, the importance of BME is supported by several studies showing that BME is a predictor of radiographic evidence of progression [5–13]. A recent study even showed that the persisting presence of BME is associated with an odds ratio (OR) of 60 for erosive progression at the same location [14].

In addition to BME, anti-citrullinated protein antibodies (ACPA) are also a strong predictor of radiographic progression [15–23]. Up to two-thirds of patients with RA harbor ACPA, as has been known for many years [24]. However, the underlying mechanism linking ACPA with a more severe disease progression with increased joint destruction is incompletely elucidated. Recent data suggest that ACPA influences bone resorption by directly activating osteoclasts [25]. The combination of these findings lead us to hypothesize that ACPA are associated with BME.

Other studies, including a small study that we performed previously suggest there is association between BME and ACPA [26, 27]. However, ACPA are often simultaneously present with other RA-related autoantibodies such as rheumatoid factor (RF) and anti-carbamylated protein antibodies (anti-CarP) (which also have been associated with radiographic destruction) [28–30]. To our knowledge the effects of different autoantibodies (either alone or in combination) on BME are unknown. Furthermore, the association between different autoantibodies and other types of inflammation detected by MRI (synovitis and tenosynovitis) has never been thoroughly explored. Therefore, this cross-sectional study aimed to investigate the associations of ACPA, RF and anti-CarP antibodies with BME, synovitis and tenosynovitis.

## Methods

### Patients

The 589 patients with early arthritis were consecutively included in the Leiden Early Arthritis Clinic (EAC) between 2010 and 2014. The EAC is an inception cohort that includes patients attending the rheumatologist who present with clinically confirmed arthritis and symptom duration of <2 years. Patients were disease-modifying-antirheumatic-drug (DMARD)-naïve at inclusion. The cohort started in 1993. MRI was performed from 2010 onwards; 598 patients underwent MRI, and 9 were excluded from analysis because no contrast agent was administered. The median interval between inclusion in the study and MRI was 1.3 weeks. Questionnaires were administered, and joint counts and blood samples were collected at baseline [31]. Baseline serum samples were tested for ACPA (anti-CCP2, Eurodiagnostica, Arnhem, the Netherlands, cutoff value ≥7 U/ml), IgM RF (as described previously, in-house ELISA [32]) and IgG anti-CarP antibodies against carbamylated fetal calf serum (FCS). Anti-CarP was determined as described previously [28]; the cutoff for positivity for anti-CarP was based on the mean plus two times the standard deviation from a set of 204 healthy controls. One year after presentation, 183 patients fulfilled the 1987 criteria for RA [33], 214 had undifferentiated arthritis (UA) and 192 had other forms of arthritis, including psoriatic arthritis, reactive arthritis and others (Table 1).

**Table 1:** Baseline characteristics of the total group of patients with early arthritis and the subgroups of patients with rheumatoid arthritis (RA) or undifferentiated arthritis (UA)

Variable	All patients with early arthritis	Subgroup of patients with RA or UA
	(n = 589)	(n = 397)
Age, mean (sd)	54.8 (16)	54.9 (15)
Female, *n* (%)	363 (62)	253 (64)
Symptom duration, median (IQR), weeks	12.6 (5–27)	12.2 (5–26.2)
TJC, median (IQR)	4 (2–7)	4 (2–6)
SJC, median (IQR)	3 (2–7)	3 (2–7)
CRP (mg/L), median (IQR)	5.7 (3–17)	6 (3–17)
ACPA positivity, *n* (%)	141 (24)	123 (31)
RF positivity, *n* (%)	193 (33)	151 (38)
anti-CarP positivity, *n* (%)	88 (15)	71 (18)
Total RAMRIS, median (IQR)	12.5 (5.5–24)	13.5 (6–24)
Total BME score, median (IQR)	2.5 (1–6)	2.5 (1–6)
Total synovitis score, median (IQR)	3.5 (1–7.5)	4 (1.5–8)
Total tenosynovitis score, median (IQR)	2 (0–6)	3 (0.5–6)

The diagnoses of the 589 patients with early arthritis were: 183 RA (according to the 1987 RA criteria), 214 UA, 14 reactive arthritis, 14 gout, 2 pseudogout, 30 psoriatic arthritis, 35 inflammatory osteoarthritis, 4 Lyme’s arthritis, 1 paramalignant arthritis, 3 systemic lupus erythematosus, 11 other systemic disorder, 7 mixed connective tissue disease, vasculitis, 4 sarcoidosis, 9 spondyloarthritis, 8 remitting seronegative symmetrical synovitis with pitting edema, and 50 other unspecified conditions. *n* number of patients, *sd* standard deviation, *IQR* interquartile range, symptom duration time between symptom onset and inclusion in cohort, *TJC* 68 tender joint count, *SJC* 66 swollen joint count, *CRP* C-reactive protein, *ACPA* anti-citrullinated protein antibodies, *RF* rheumatoid factor, *anti-CarP* anti-carbamylated protein antibodies, *RA*/*UA* subgroup of patients with rheumatoid arthritis (according to the 1987 RA criteria) or undifferentiated arthritis

### Magnetic resonance imaging and scoring

At baseline, MRI was performed of the metacarpophalangeal (MCP), wrist and metatarsophalangeal (MTP) joints on the most painful side or on the dominant side in the case of symmetric symptoms. MRI was performed using an MSK Extreme 1.5 T extremity MRI system. In the wrist and MCP joints a coronal T1-weighted sequence was acquired before intravenous injection of contrast agent (gadoteric acid). Post-contrast, coronal and axial T1-weighted sequences with frequency-selective fat saturation were obtained. The forefoot was scanned using a T1-weighted sequence and a T2-weighted fat-saturated sequence in the axial plane. The protocol was shortened after 371 patients had been imaged [34]. For post-contrast imaging of the foot in the remaining 218 patients, T1-weighted, fat-saturated sequences were obtained in the coronal and axial plane and the T2-weighted sequence was deleted. A more detailed description of the scan protocol is provided elsewhere [14, 35, 36] and in Additional file 1.

BME and synovitis were scored semi-quantitatively according to the rheumatoid arthritis MRI scoring system (RAMRIS) [37], with the exception that BME was assessed on a contrast-enhanced T1-weigthed fat-suppressed sequence. Previous studies have shown that T2-weighted fat-saturated sequences and contrast-enhanced T1-weighted fat-saturated images perform equally well in the depiction of BME [34, 38, 39], and according to the European Society of Musculoskeletal Radiology (ESSR), both sequences can be used to evaluate BME [40]. The T1-sequence was used as it allowed a shorter scan time. In addition, tenosynovitis in the wrist and MCP joints was scored according to the method proposed by Haavardsholm et al. [41], with tenosynovitis assessed for the flexor and extensor tendons of MCP joints 2–5 using the same scale of 0–3 as for the wrist. MR images were scored by two readers blinded to any clinical data. The mean total scores of both readers for BME, synovitis and tenosynovitis were used in further analyses. The intra-reader class correlation coefficients for the total inflammation score based on 40 MR images that were scored twice, were 0.98 and 0.93, respectively. Based on all 598 scans, the inter-reader class correlation coefficient for the total inflammation score was 0.95.

### Sensitivity analyses

Our primary analyses were performed in all 589 early arthritis patients, as we hypothesized that direct association between autoantibodies and MRI-detected inflammation, if present, would be independent of the clinical diagnosis. However, analyses were repeated in the subgroup of 397 patients classified with UA or RA according to the 1987 American College of Rheumatology (ACR) criteria. Patients who had UA after one year were included in these analyses because misclassification could have occurred due to DMARD treatment during the first year that could have hampered progression to fulfilling the 1987 criteria for RA. As some of these patients with UA would have progressed to RA without treatment (but now remain unclassified), we also studied the patients with UA.

### Statistical analysis

The *t* test, multivariable linear regression, and multivariable logistic regression were used for analysis as appropriate. In multivariable linear regression analysis, the BME scores were log_10_-transformed (log_10_(score + 1)) to approximate a normal distribution. For interpretation, the obtained effect size (beta) was back-transformed to the normal score. All models were adjusted for age, gender and symptom duration. Baseline data on ACPA, RF, and anti-CarP were dichotomized (seropositive vs. seronegative). Anti-CarP data were missing for 16 patients. ACPA and RF status was known for all patients. To determine the effect of ACPA levels on BME, baseline ACPA was categorized into three groups within ACPA-positive patients based on the range of ACPA values (low, intermediate, or high); the thresholds were: ≥7 U/ml, ≥167 U/ml and ≥327 U/ml. *P* values ≤0.05 were considered significant. Analyses were performed using SPSS version 23.0 (IBM).

## Results

### Baseline characteristics

Baseline characteristics of the 589 patients are presented in Table 1.

### ACPA is associated with BME at baseline

**Fig. 1:**
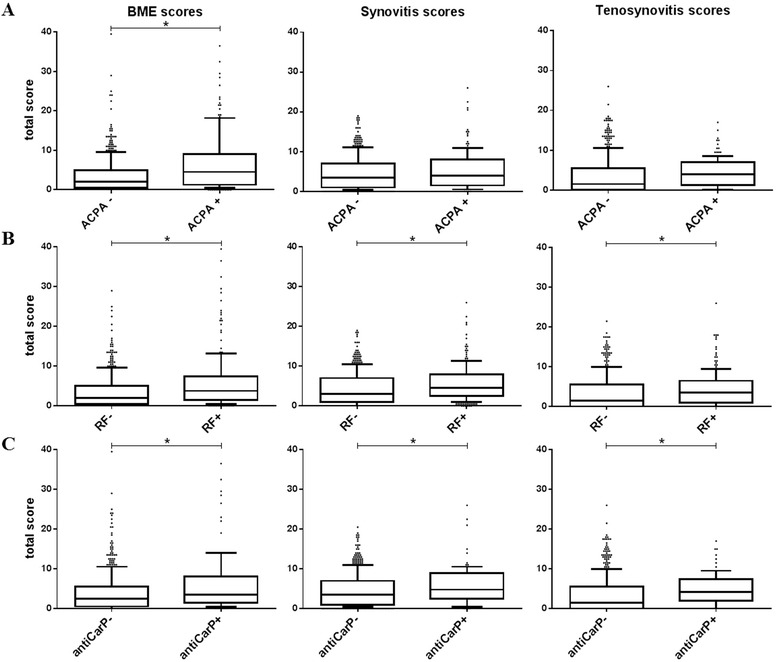
Illustration of the association between anti-citrullinated protein antibodies (*ACPA*) (**a**), rheumatoid factor (*RF*) (**b**) and anti-carbamylated protein antibodies (*anti-CarP*) (**c**), and magnetic-resonance-imaging-detected bone marrow edema (*BME*), synovitis and tenosynovitis scores in early arthritis (n = 589). *Horizontal lines* represent median. *Whiskers* show the 10^th^–90^th^ percentile. *Dots* represent outliers. **a** BME: *p* ≤ 0.001; synovitis: *p* = 0.084; tenosynovitis: *p* = 0.064. **b** BME: *p* ≤ 0.001; synovitis: *p* = 0.001; tenosynovitis: *p* = 0.004. **c** BME: *p* ≤ 0.012; synovitis: *p* = 0.021; tenosynovitis: *p* = 0.013. Total score: sum of scores in metacarpophalangeal, wrist, and metatarsophalangeal joints. *Significant difference (*p* < 0.05) between autoantibody-negative and autoantibody-positive patients

We first evaluated whether patients with ACPA (n = 141) or without ACPA (n = 448) had differences in BME scores (Fig. 1a). ACPA-positive patients had higher BME scores (median = 4.5) than ACPA-negative patients (median = 2.0, *p* < 0.001). We subsequently questioned whether ACPA is also associated with other types of MRI-detected inflammation, i.e., synovitis and tenosynovitis. There were no statistically significant differences in synovitis or tenosynovitis scores in ACPA-positive and ACPA-negative patients (Fig. 1a). Similar results were obtained for BME when only patients with RA and UA were studied (ACPA-positive median = 3.5, ACPA-negative median = 2.0, *p* = 0.001) and no statistically significant differences were observed for synovitis and tenosynovitis (Additional file 2: Figure S1A). Based on these data ACPA seemed to be primarily associated with BME.

### RF and anti-CarP antibodies are also associated with BME

We were also interested in whether RF and anti-CarP antibodies are also associated with more severe BME. The BME scores were higher in RF-positive patients (median = 3.75) compared to RF-negative patients (median = 2.0, *p* < 0.001, Fig. 1b). Similarly, BME scores were also higher in anti-CarP-positive than in anti-CarP-negative patients (median = 3.5 vs. 2.5, *p* = 0.012, Fig. 1c).

Besides BME, synovitis and tenosynovitis scores were also higher in RF-positive than in RF-negative patients (synovitis: median 4.5 vs. 3.0, p = 0.001; tenosynovitis: median 3.5 vs. 1.5, *p* = 0.004). Synovitis and tenosynovitis scores were also higher in anti-CarP-positive than in anti-CarP-negative patients (synovitis: median = 4.75 vs. 3.5, *p* = 0.021; tenosynovitis: median = 4.25 vs. 1.5, *p* = 0.013). In patients with RA and UA only the BME scores were significantly higher in RF-positive (RF+) or anti-CarP-positive (anti-CarP+) patients, but synovitis and tenosynovitis scores were not statistically significantly different (BME: RF+ median = 3.5, RF-negative (RF-) median = 2.0, *p* = 0.002; anti-CarP+ median = 3.5, anti-CarP-negative (anti-CarP-) median = 2.5, *p* = 0.017, Additional file 2: Figure S1B, C).

Patients can concurrently have BME, synovitis, and tenosynovitis. To unravel the independent association between RF and BME, synovitis, and tenosynovitis scores, multivariable logistic regression analysis was performed with RF as the dependent variable and BME, synovitis, and tenosynovitis as independent variables. The same was done with anti-CarP as the dependent variable. In early arthritis, only the BME score was independently associated with RF (*p* < 0.001) or with anti-CarP (*p* = 0.003). Similar results were observed in the subgroup of patients with RA or UA, in whom only BME was associated with RF (*p* < 0.001) or with anti-CarP (*p* = 0.001). Thus, these multivariable analyses suggest that the BME score is independently associated with RF or anti-CarP, in contrast to the synovitis and tenosynovitis scores. Because of this result, and because it was observed that there was an association between BME and ACPA, subsequent analyses focused on BME.

### ACPA and RF are both independently associated with BME

Patients frequently have a combination of different types of inflammation, and also concomitantly have the three autoantibodies. For more insight into the relationship between the different autoantibodies and BME, multivariable linear regression analysis was performed with BME as the outcome and the three autoantibodies as independent variables. Both ACPA and RF were significantly associated with BME (ACPA: *p* = 0.015, beta = 1.33, indicating that ACPA-positive (ACPA+) patients had 33 % higher BME scores than ACPA-negative (ACPA-) patients; RF: *p* = 0.004, beta = 1.31, indicating that RF+ patients had 31 % higher BME scores than RF- patients). Additional adjustments for CRP and SJC produced similar results (ACPA: *p* = 0.009, beta = 1.36; RF: *p* = 0.001, beta = 1.36). In a similar analysis in the subgroup of patients with RA and UA, there was a trend towards significance for ACPA (*p* = 0.091, beta = 1.26) and a significant result for RF (RF: *p* = 0.022, beta = 1.31). Thus, together these data indicated that ACPA and RF are independently associated with BME scores.

### Combined presence of ACPA and RF is associated with BME

**Fig. 2:**
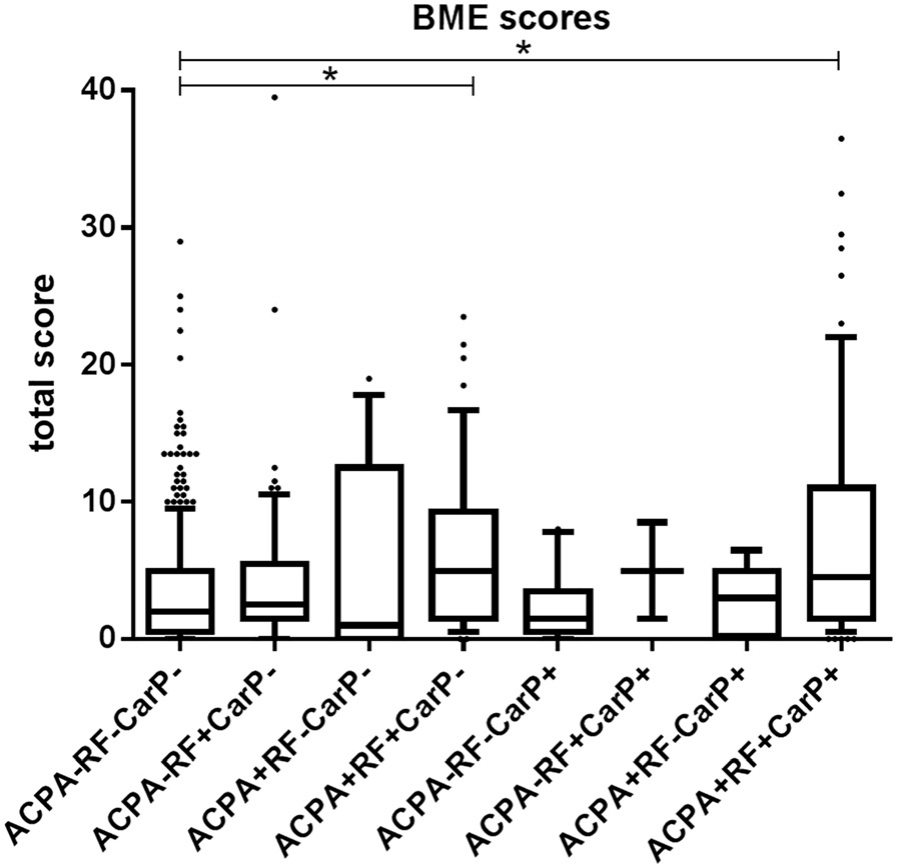
Bone marrow edema (BME) scores in patients with early arthritis (n = 589) with different combinations of anti-citrullinated protein antibodies (ACPA), rheumatoid factor (RF) and anti-carbamylated protein antibodies (anti-CarP). Horizontal lines represent median. Whiskers show the 10^th^–90^th^ percentile. Dots represent outliers. ACPA+, RF+, anti-CarP- patients vs. ACPA-, RF-, anti-CarP- patients, *p* < 0.001; ACPA+, RF+, anti-CarP+ patients vs. ACPA-, RF-, anti-CarP- patients, *p* < 0.001. ACPA-, RF-, anti-CarP- patients, n = 353; ACPA-, RF+, anti-CarP- patients, n = 68; ACPA+, RF-, anti-CarP- patients, n = 15; ACPA+, RF+, anti-CarP- patients, n = 48; ACPA-, RF-, anti-CarP+ patients, n = 11; ACPA-, RF+, anti-CarP+ patients, n = 3; ACPA+, RF-, anti-CarP+ patients, n = 5; ACPA+, RF+, anti-CarP+ patients, n = 69. Total score: sum of BME scores in metacarpophalangeal, wrist, and metatarsophalangeal joints. *Significant difference (*p* < 0.05) between subgroups

**Fig. 3:**
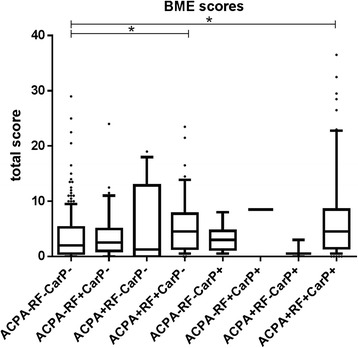
Bone marrow edema (BME) scores in patients with rheumatoid arthritis (RA) and undifferentiated arthritis (UA) (n=397) with different combinations of anti-citrullinated protein antibodies (ACPA), rheumatoid factor (RF) and anti-carbamylated protein antibodies (anti-CarP). Horizontal lines representing median. Whiskers show the 10^th^–90^th^ percentile. Dots represent outliers. ACPA+, RF+, anti-CarP+ patients vs. ACPA-, RF-, anti-CarP- patients, *p* < 0.001; ACPA+, RF+, anti-CarP- patients vs. ACPA-, RF-, anti-CarP- patients, *p* < 0.001. ACPA-, RF-, anti-CarP- patients, n = 217; ACPA-, RF+, antiCarP- patients, n = 43; ACPA+, RF-, anti-CarP- patients, n = 14; ACPA+, RF+, anti-CarP- patients, n = 42; ACPA-, RF-, anti-CarP+ patients, n = 6; ACPA-, RF+, anti-CarP+ patients, n = 1; ACPA+, RF-, anti-CarP+ patients, n = 3; ACPA+, RF+, anti- CarP+ patients, n = 61. Total score: sum of BME scores in metacarpophalangeal, wrist, and metatarsophalangeal joints. *Significant difference between subgroups (*p* < 0.05)

The multivariable analyses described above did not evaluate different effects for combinations of antibodies. Different autoantibody combinations were compared for more insight into the effect of individual antibodies and a combination of antibodies on BME (Fig. 2). In the absence of both RF and anti-CarP, ACPA was not associated with BME (ACPA+, RF-, anti-CarP- patients vs. ACPA-, RF-, anti-CarP- patients, median 1.0 vs. 2.0, *p* = 0.43). Also the presence of RF or anti-CarP alone was not associated with BME (ACPA-, RF+, anti- CarP- patients and ACPA-, RF-, anti-CarP+ patients vs. ACPA-, RF-, anti-CarP- patients, median 2.5 and 1.5 vs 2.0 respectively, *p* = 0.096 and *p* = 0.43). However ACPA+, RF+, anti-CarP- patients and ACPA+, RF+, anti-CarP+ patients did have significantly higher BME-scores than ACPA-, RF-, anti-CarP- patients (median 5.0 and 4.5 vs. 2.0 respectively, *p* < 0.001 and *p* < 0.001). The same analysis in only RA- and UA-patients showed that ACPA+, RF+, anti-CarP- patients and ACPA+, RF+, anti-CarP+ patients had higher BME-scores than ACPA-, RF-, anti-CarP- patients (median 4.5 and 4.5 vs. 2.0 respectively, *p* < 0.001 and *p* < 0.001, Fig. 3). Thus only the combined presence of ACPA and RF (with or without the presence of anti-CarP) was associated with higher BME-scores.

### ACPA level is not associated with BME

**Fig. 4:**
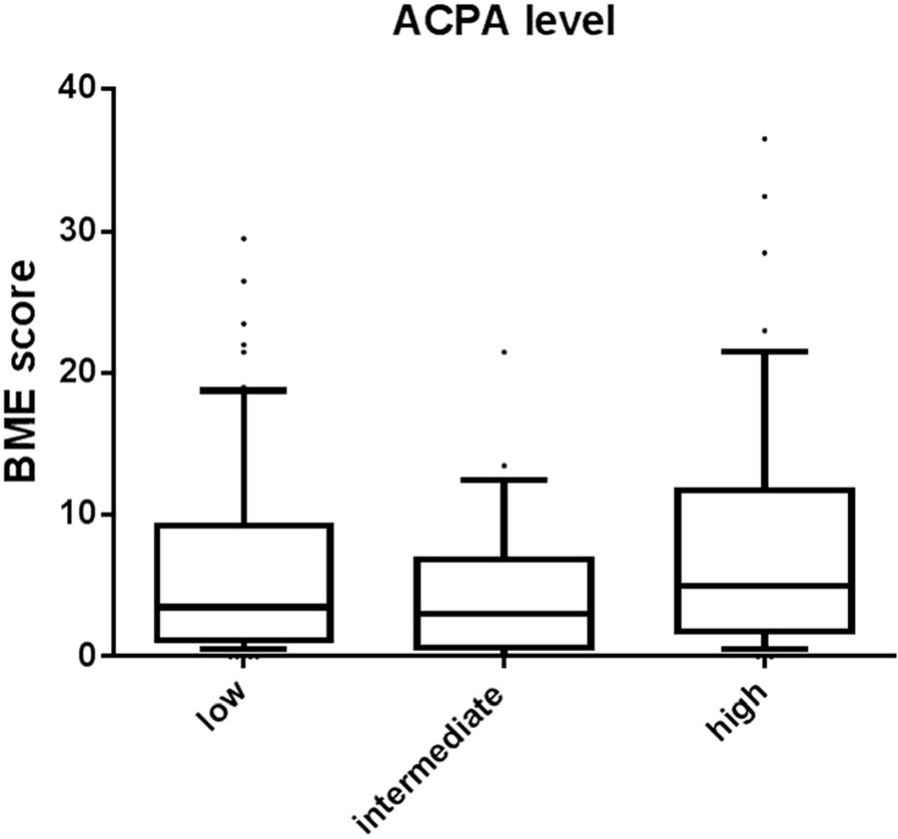
Bone marrow edema (*BME*) scores in anti-citrullinated protein antibodies (*ACPA*)-positive patients with early arthritis (n = 141) with low, intermediate, or high levels of ACPA. *Horizontal lines* represent median. *Whiskers* show the 10^th^–90^th^ percentile. *Dots* represent outliers. Baseline ACPA is shown categorically as low, intermediate, or high. The groups were as follows: low ≥7 U/ml, intermediate ≥167 U/ml and high ≥327 U/ml. Low: n = 64; intermediate: n = 32; high: n = 45. Kruskal-Wallis test, *p* = 0.14

In general, patients who carry different RA-related autoantibodies also have higher levels of ACPA [42]. In our present data we also observed higher ACPA levels in patients who also carried RF and anti-CarP (ACPA+, RF-, anti-CarP- patients median 116 U/ml, ACPA+, RF+, anti-CarP- patients median 155 U/ml, ACPA+, RF-, anti-CarP+ patients median 92 U/ml, ACPA+, RF+, anti-CarP+ patients median 257 U/ml, *p* = 0.020). This prompted us to explore whether the combined presence of ACPA and RF with higher BME scores could be explained by higher ACPA levels. To investigate the association between BME and ACPA levels, ACPA were studied as continuous data (Additional file 3: Figure S2) and divided into three subgroups. The BME scores observed in these ACPA categories were not different (Fig. 4). Similarly, no differences were observed when analyzing the BME scores in relation to ACPA levels in patients with RA and UA (Additional file 4: Figure S3). These data suggest that it is the combined presence of ACPA and RF that is associated with BME, rather than ACPA levels.

### Combined presence of ACPA, RF and anti-CarP is associated with synovitis and tenosynovitis

**Fig. 5:**
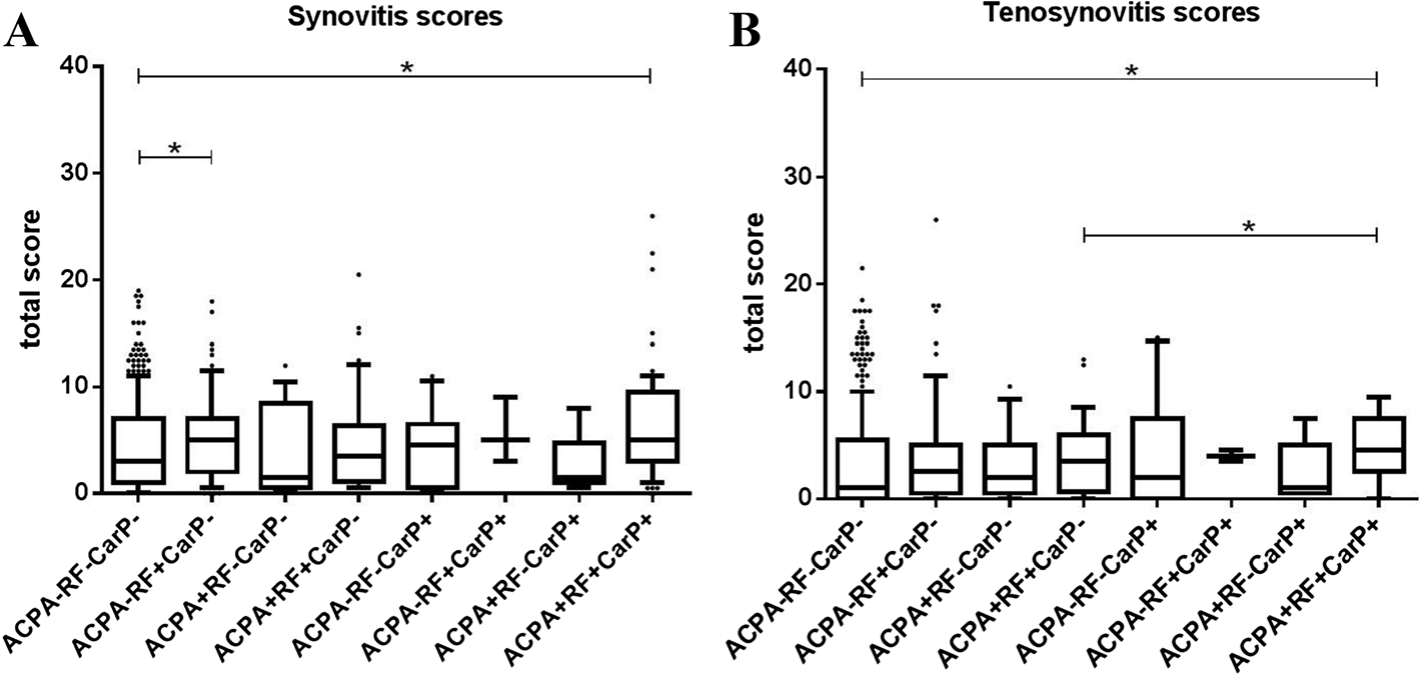
Scores for synovitis detected by magnetic resonance imaging (**a**) and tenosynovitis (**b**) in patients with early arthritis (n = 589) with different combinations of anti-citrullinated protein antibodies (ACPA), rheumatoid factor (RF) and anti-carbamylated protein antibodies (anti-CarP). Horizontal lines represent median. Whiskers show the 10^th^–90^th^ percentile. Dots represent outliers. Synovitis: ACPA+, RF+, anti-CarP+ patients vs. ACPA-, RF-, anti-CarP- patients, *p* < 0.001. Tenosynovitis: ACPA+, RF+, anti-CarP+ patients vs. ACPA-, RF-, anti-CarP- patients, *p* < 0.001; ACPA+, RF+, anti-CarP+ patients vs. ACPA+, RF+, anti-CarP- patients, *p* = 0.039. ACPA-, RF-, anti-CarP- patients, n = 353; ACPA-, RF+, anti-CarP- patients, n = 69; ACPA+, RF-, anti-CarP- patients, n = 15; ACPA+, RF+, anti-CarP- patients, n = 48; ACPA-, RF-, anti-CarP+ patients, n = 11; ACPA-, RF+, anti-CarP+ patients, n = 3; ACPA+, RF-, anti-CarP+ patients, n = 5; ACPA+, RF+, anti-CarP+ patients, n = 69. Total score: sum of scores in metacarpophalangeal, wrist, and metatarsophalangeal joints. *Significant difference between subgroups (*p* < 0.05)

The analyses focused on BME as the different autoantibodies were not associated with synovitis or tenosynovitis scores in univariable analyses (for ACPA) or in multivariable analyses (for RF and anti-CarP). However, having observed that higher BME scores were primarily associated with the combined presence of ACPA and RF (and not with the presence of a single antibody), we reasoned that it might also be possible that antibodies were not individually associated with synovitis or tenosynovitis scores, but that some combinations of autoantibodies were associated with synovitis or tenosynovitis scores. To study this, we finally assessed the association between different combinations of autoantibodies and synovitis and tenosynovitis (Fig. 5). ACPA+, RF+, anti-CarP+ patients had higher synovitis scores than ACPA-, RF-, anti-CarP- patients (median 5.0 vs. 3.0, *p* = 0.001). For tenosynovitis, ACPA+, RF+, anti-CarP+ patients had higher scores than ACPA-, RF-, anti-CarP- patients (median 4.5 vs. 1.0, *p* < 0.001), and ACPA+, RF+, anti-CarP+ patients had higher scores than ACPA+, RF+, anti-CarP- patients (median 4.5 vs. 3.5 *p* = 0.039). Thus, the combined presence of ACPA, RF and anti-CarP was associated with the highest synovitis and tenosynovitis scores.

## Discussion

The relationship between ACPA and other RA-related autoantibodies and BME was subject of this study. We showed that ACPA, RF and anti-CarP antibodies were all associated with BME in univariable analyses. However, when the different autoantibody combinations were compared, the presence of ACPA alone was not associated with BME, but the combined presence of ACPA and RF (with or without anti-CarP) was associated with BME. The level of ACPA was not associated with BME, suggesting that this cannot be explained by ACPA levels but rather by the combined presence of ACPA and RF.

To our knowledge this is the first study including almost 600 MR images in which the relationship between the different autoantibodies and MRI-detected inflammation was investigated in detail. Due to this large sample size it was possible to evaluate the independent associations between BME and ACPA, RF and anti-CarP. Furthermore, it was possible to investigate the differential effects of the autoantibodies on the different types of MRI-detected inflammation. On analyses in subgroups of patients with different autoantibody combinations the BME scores were mainly increased when both ACPA and RF were present.

Our data suggest a potential interaction between RF and ACPA; however, the underlying mechanism by which ACPA and RF could act in concert was not studied. Potentially RF could have an immune-enhancing effect by crosslinking immune complexes and thereby activate monocytes or macrophages and induce cytokine expression. This is supported by a recent study that showed that RF augments TNFα production by ACPA immune complexes in vitro [43]. Another explanation could be that RF has a role in immune complex stabilization. ACPA bind to target antigens with low avidity but it could well be that when RF is also involved in the immune complex this binding is more stable. Further fundamental studies should be performed for more insight into the interaction between these two autoantibodies.

This study investigated local inflammation as observed on MRI. Recently the combined effect of ACPA and RF on systemic inflammation was investigated in RA, showing that the combined presence of ACPA and RF was associated with higher levels of pro-inflammatory cytokines and increased acute phase reactants and disease activity [43]. We also analyzed the association between the different autoantibody combinations and CRP, erythrocyte sedimentation rate (ESR), SJC and 28-joint-count disease activity score (DAS28) as measures of disease activity in our patients with RA or UA at baseline; no large differences were observed but patients positive for all three autoantibodies had the highest disease activity scores (Additional file 5: Figure S4).

Association between the combined presence of autoantibodies and BME was observed. Since BME is associated with erosive progression [5–14], it would be interesting to investigate whether combinations of autoantibodies are also associated with radiographic progression. The association between ACPA or RF and radiographic progression is well-investigated [15–23]. However the number of studies investigating the combined effect of ACPA and RF is limited. A recent study in two cohorts showed no additive effect of RF on radiographic progression in ACPA-positive patients [44]. Another study analyzing high-resolution peripheral quantitative computed tomography (CT) images of the MCP joints in patients with RA showed that there was an additive effect of ACPA and RF on erosion number and size [45]. The read-out of these studies (microCT and conventional radiography) was different. It would be interesting to further unravel the association between different autoantibody combinations and erosive progression in further observational studies.

A limitation of the subgroup analysis is that some autoantibody combinations were infrequent and so no definite conclusions can be drawn for these. For instance, patients who were ACPA+, RF-, anti-CarP- were infrequent. Despite the limited power, there was no tendency in the data towards higher BME scores in these patients compared to the triple-negative group. This study is not the first that did not identify a deleterious effect of the presence of ACPA alone. Two recent papers reported on mice that were injected with ACPA, and although ACPA was detected in the joint, no signs of inflammation were observed in the synovium [46, 47]. Surprisingly, in our data the presence of ACPA alone even had a non-significant tendency towards a protective effect against synovitis (in all patients with early arthritis patients and in patients with RA or UA). Interestingly, two recent studies in humans showed that the presence of ACPA without RF was associated with lower disease activity [42, 43]. In summary, further larger studies are needed to determine the role of ACPA single positivity.

Another limitation could be that we used contrast-enhanced T1-weighted images to assess BME. Using the RAMRIS method, T2-weighted fat-suppressed sequences, or when this sequence is not available, a short tau inversion recovery (STIR) sequence, should be used to assess BME. However, it has been demonstrated that a contrast-enhanced T1-weighted fat-suppressed sequence performed equally well to depict BME as a T2-weighted fat-suppressed sequence [34, 38, 39] and the evaluation of BME on a T1-weighted fat suppressed sequence is also supported by the ESSR [40]. In this study the contrast-enhanced T1-weighted fat-suppressed sequence was used because it allowed a shorter scan time and has a higher signal-to-noise ratio [34, 38].

A third limitation is that the scan protocol for the foot was changed. When the analyses of the different autoantibody combinations and BME scores in patients with early arthritis were repeated separately in the patients scanned with or without the coronal sequence of the foot, the presence of ACPA alone was not associated with higher BME scores, but the combined presence of ACPA and RF was associated with higher BME scores (data not shown). This suggests that the change in scan protocol for the foot had no major influences on the results of this study.

Finally, our arthritis cohort includes patients with early disease who presented with different diagnoses. We hypothesized that direct association between ACPA and MRI-detected inflammation would be independent of the clinical diagnosis. However, to exclude an effect of this heterogeneity in patient selection on our findings we repeated all analyses within the subgroup of patients with RA and UA. This produced similar results.

Of note, the differences observed in BME scores were statistically significant but the absolute differences were relatively small. The variation in BME scores was only partly explained by the autoantibody status. Nonetheless the present study does increase our understanding of the relationship between autoantibodies and BME, which are both predictors of radiographic progression. The observation that ACPA is associated with osteitis, only when RF is present, fuels further laboratory studies on the biological relevance of these autoantibodies.

## Conclusions

In conclusion, the presence of ACPA alone and ACPA serum levels were not associated with BME scores. However, BME scores were higher when patients were seropositive for both ACPA and RF. These results suggest that RF has an additive role to ACPA in mediating osteitis.

## Abbreviations

ACPA, anti-citrullinated protein antibodies; anti-CarP, anti-carbamylated protein antibodies; BME, bone marrow edema; CRP, C-reactive protein; DMARD, disease-modifying-antirheumatic-drug; EAC, Early Arthritis Clinic; ELISA, enzyme-linked immunosorbent assay; ESR, erythrocyte sedimentation rate; ESSR, European Society of Musculoskeletal Radiology; FCS, fetal calf serum; IQR, interquartile range; MCP, metacarpophalangeal; MRI, magnetic resonance imaging; MTP, metatarsophalangeal ; OR, odds ratio; RA, rheumatoid arthritis; RAMRIS, rheumatoid arthritis magnetic resonance imaging scoring system; RF, rheumatoid factor; SD, standard deviation; SJC, swollen joint count; TJC, tender joint count; TNF, tumor necrosis factor; UA, undifferentiated arthritis

## Additional files

Additional file 1: MRI protocol. (DOC 36 kb) Additional file 2: Figure S1. Illustration of association between ACPA (**A**), RF (**B**) and anti-CarP (**C**) and MRI-detected BME, synovitis and tenosynovitis scores in patients with RA and UA (n = 397). *Horizontal lines* represent median. *Whiskers* show the 10^th^–90^th^ percentile. *Dots* represent outliers. **A** BME: *p* = 0.001; synovitis: *p* = 0.776; tenosynovitis: *p* = 0.99. **B** BME: *p* = 0.002; synovitis: *p* = 0.19; tenosynovitis: *p* = 0.26. **C** BME: *p* = 0.017; synovitis: *p* = 0.085; tenosynovitis: *p* = 0.056. Total score: sum of scores in MCP, wrist, and MTP joints. *MRI* magnetic resonance imaging, *ACPA* anti-citrullinated protein antibodies, *RF* rheumatoid factor, *anti-CarP* anti-carbamylated protein antibodies, *BME* bone marrow edema. *Significant difference between autoantibody-negative and autoantibody-positive patients (*p* < 0.05). (JPG 76 kb) Additional file 3: Figure S2. Association between ACPA level and BME scores. Association between ACPA level and BME scores within ACPA-positive patients with early arthritis (**A**) (n = 141, *r* = 0.071, *p* = 0.403) and within ACPA-positive patients with RA or UA (**B**) (n = 123, *r* = 0.034, *p* = 0.706). (JPG 24 kb) Additional file 4: Figure S3. BME scores of ACPA-positive patients with RA or UA (n = 123) with low, intermediate, or high levels of ACPA. *Horizontal lines* represent median. *Whiskers* show the 10^th^–90^th^ percentile. *Dots* represent outliers. Baseline ACPA levels are shown categorically as low, intermediate, or high. The groups were as follows: low ≥7 U/ml, intermediate ≥167 U/ml, and high ≥327 U/ml. Low: n = 57; intermediate: n = 27; high: n = 39. *ACPA* anti-citrullinated protein antibodies, *BME* bone marrow edema. Kruskal-Wallis test, *p* = 0.23. (JPG 19 kb) Additional file 5: Figure S4. Measures of disease activity in patients with RA and UA (n = 397) with different combinations of ACPA, RF and anti-CarP. Association between CRP (**A**), ESR (**B**), SJC (**C**) and DAS28 (**D**) and different autoantibody combinations. *Horizontal lines* represent median. *Whiskers* show the 10^th^–90^th^ percentile. *Dots* represent outliers. *ACPA* anti-citrullinated protein antibodies; RF: rheumatoid factor; anti-CarP: anti-carbamylated protein antibodies, *CRP* C-reactive protein, *ESR* erythrocyte sedimentation rate, *SJC* swollen joint count based on 66 joints, *DAS28* disease activity score in 28 joints. *Significant difference between subgroups (*p* < 0.05). (JPG 104 kb)
