# Supplementary material for: MRI-detected osteitis is not associated with the presence or level of ACPA alone, but with the combined presence of ACPA and RF
Source: Arthritis Res Ther. 2016 Aug 2;18:179. doi: 10.1186/s13075-016-1076-0 (PMC4971651; doi:10.1186/s13075-016-1076-0)
Supplement: Additional file 1: — MRI protocol. (DOC 36 kb) [file 13075_2016_1076_MOESM1_ESM.doc]

Additional file 1

Detailed MRI protocol

MR imaging was performed on a MSK-extreme 1.5T extremity MR imaging system (GE, Wisconsin, USA) using a 145mm coil for the foot and a 100mm coil for the hand. The patient was positioned in a chair beside the scanner, with the hand or foot fixed in the coil with cushions.

In the hand (MCP2-5 and wrist) the following sequence was acquired before contrast administration: T1-weighted fast spin-echo (FSE) sequence in the coronal plane (repetition time (TR) 575 ms, echo time (TE) 11.2 ms, acquisition matrix 388×288, echo train length (ETL) 2). After intravenous injection of gadolinium contrast (gadoteric acid, Guerbet, Paris, France, standard dose of 0.1 mmol/kg) the following sequences were obtained: T1-weighted FSE sequence with frequency selective fat saturation (fatsat) in the coronal plane (TR/TE 700/9.7ms, acquisition matrix 364×224, ETL 2), T1-weighted FSE sequence with frequency selective fat saturation in the axial plane (wrist: TR/TE 540/7.7 ms; acquisition matrix 320x192; ETL 2 and MCP-joints: TR/TE 570/7.7 ms; acquisition matrix 320x192; ETL 2).

The obtained sequences of the forefoot (MTP1-5 joints) were for the first 371 patients before contrast administration: T1-weighted FSE sequence in the axial plane (TR/TE 650/17ms; acquisition matrix 388x288, ETL 2); and T2-weighted FSE fatsat sequence in the axial plane (TR/TE 3000/61.8; acquisition matrix 300x224, ETL 7). Imaging of the foot was initially limited to pre-contrast axial sequences. For the later 218 patients post-contrast sequences were included: T1-weighted FSE fatsat sequence in the axial plane (TR/TE 700/9.5ms; acquisition matrix 364x224, ETL 2) and T1-weighted FSE fatsat sequence in the coronal plane (perpendicular to the axis of the metatarsals) (TR/TE 540/7.5ms; acquisition matrix 320x192, ETL 2).

Field-of-view was 100mm for the hand and 140mm for the foot. Coronal sequences of the hand had 18 slices with a slice thickness of 2mm and a slice gap of 0.2mm. Coronal sequences of the foot had 20 slices with a slice thickness of 3mm and a slice gap of 0.3mm. All axial sequences had a slice thickness of 3mm and a slice gap of 0.3mm with 20 slices for the wrist, 16 for the metacarpophalangeal-joints and 14 for the foot.

According to the RAMRIS-method, T2-weighted fat suppressed sequences, or when this sequence is not available a short tau inversion recovery (STIR) sequence, should be used to assess BME. Previously, three studies have demonstrated that a contrast enhanced T1-weighted fat suppressed sequence has a strong correlation with T2-weighted fat suppressed sequences [1–3]. A T2-weighted image shows increased water signal and a contrast-enhanced T1-weighted sequence shows increased water content and the increased perfusion and interstitial leakage. A strong correlation has been shown in arthritis patients but also in patients without inflammatory diseases such as bone bruises, intraosseous ganglions, bone infarcts and even nonspecific cases [2,3]. We used the contrast enhanced T1-weighted fat suppressed sequence as it allowed a shorter scan time and has a higher signal to noise ratio.

MRI scoring and dichotomizing

All bones, joints and tendons were scored semi-quantitatively according to the validated RA MRI scoring system (RAMRIS). BME was scored on a 0-3 scale based on the affected volume of the bone (no BME, >0-33%, >33-66%, >66%), the synovitis-score (range 0-3) was scored based on the volume of enhancing tissue in the synovial compartment (none, mild, moderate, severe) and the tenosynovitis-score (ranged 0-3) was based on the thickness of peritendinous effusion or synovial proliferation with contrast enhancement (normal, <2mm, 2-5mm, >5mm) [4,5]. The scores of all joints were summed.

Missing scores; infrequently BME, synovitis or tenosynovitis could not be reliably assessed. This was mostly due to inhomogeneous fat suppression or movement artifacts. In total: 946 (2.4%) individual BME scores 339 (2.4%), synovitis scores and 169 (0.8%) tenosynovitis scores were missing. Missingness was considered to be completely at random. In order to prevent that the total MRI should be disregarded, these missing values were imputed with the median value for that feature across all locations within the same patient of that scorer.
