# Supplementary figures and images for: MRI-detected osteitis is not associated with the presence or level of ACPA alone, but with the combined presence of ACPA and RF
Source: Arthritis Res Ther. 2016 Aug 2;18:179. doi: 10.1186/s13075-016-1076-0 (PMC4971651; doi:10.1186/s13075-016-1076-0)

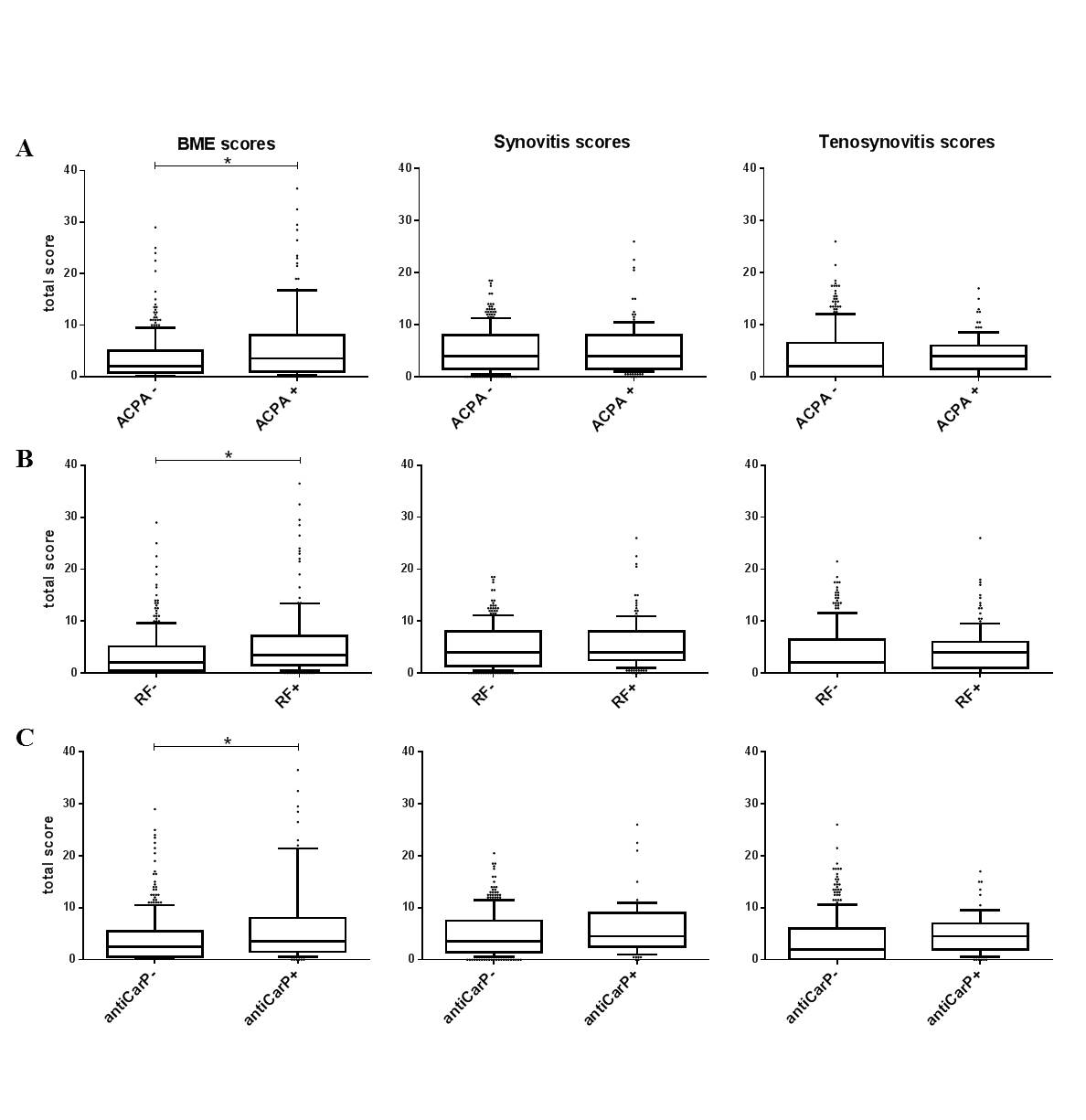

Supplement: Additional file 2: Figure S1. — Illustration of association between ACPA (A), RF (B) and anti-CarP (C) and MRI-detected BME, synovitis and tenosynovitis scores in patients with RA and UA (n = 397). Horizontal lines represent median. Whiskers show the 10th–90th percentile. Dots represent outliers. A BME: p = 0.001; synovitis: p = 0.776; tenosynovitis: p = 0.99. B BME: p = 0.002; synovitis: p = 0.19; tenosynovitis: p = 0.26. C BME: p = 0.017; synovitis: p = 0.085; tenosynovitis: p = 0.056. Total score: sum of scores in MCP, wrist, and MTP joints. MRI magnetic resonance imaging, ACPA anti-citrullinated protein antibodies, RF rheumatoid factor, anti-CarP anti-carbamylated protein antibodies, BME bone marrow edema. *Significant difference between autoantibody-negative and autoantibody-positive patients (p < 0.05). (JPG 76 kb) [file 13075_2016_1076_MOESM2_ESM.jpg]

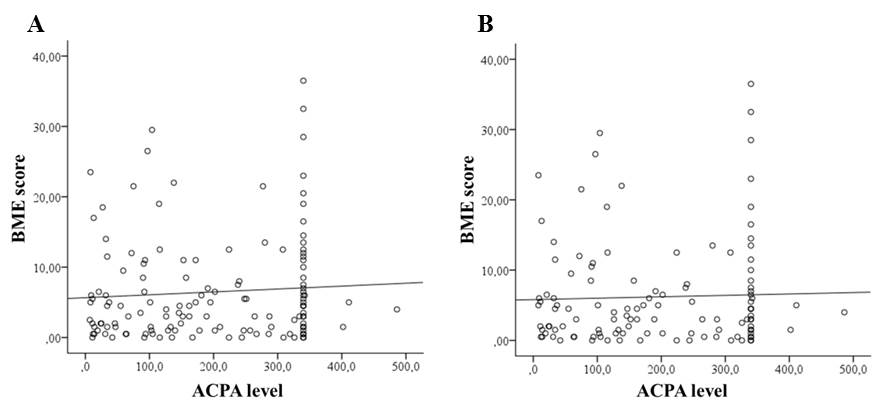

Supplement: Additional file 3: Figure S2. — Association between ACPA level and BME scores. Association between ACPA level and BME scores within ACPA-positive patients with early arthritis (A) (n = 141, r = 0.071, p = 0.403) and within ACPA-positive patients with RA or UA (B) (n = 123, r = 0.034, p = 0.706). (JPG 24 kb) [file 13075_2016_1076_MOESM3_ESM.jpg]

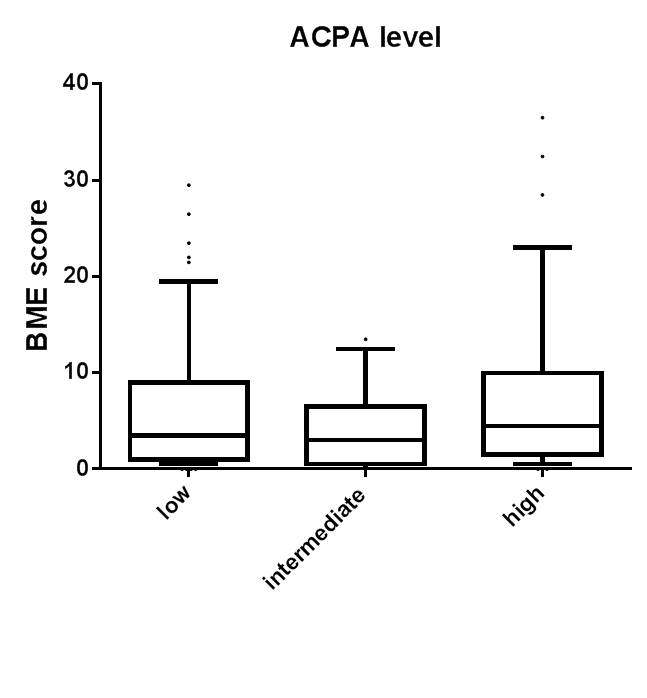

Supplement: Additional file 4: Figure S3. — BME scores of ACPA-positive patients with RA or UA (n = 123) with low, intermediate, or high levels of ACPA. Horizontal lines represent median. Whiskers show the 10th–90th percentile. Dots represent outliers. Baseline ACPA levels are shown categorically as low, intermediate, or high. The groups were as follows: low ≥7 U/ml, intermediate ≥167 U/ml, and high ≥327 U/ml. Low: n = 57; intermediate: n = 27; high: n = 39. ACPA anti-citrullinated protein antibodies, BME bone marrow edema. Kruskal-Wallis test, p = 0.23. (JPG 19 kb) [file 13075_2016_1076_MOESM4_ESM.jpg]

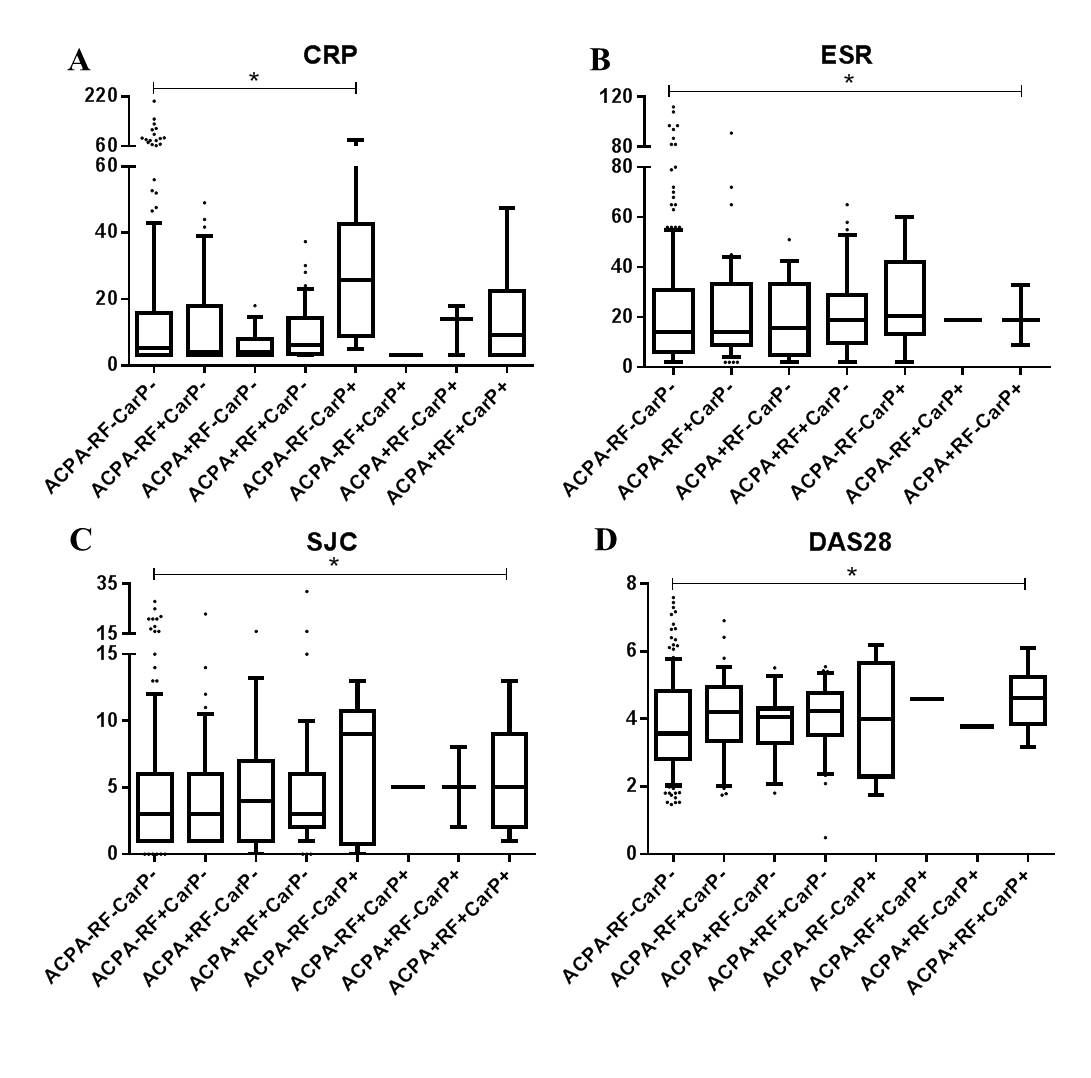

Supplement: Additional file 5: Figure S4. — Measures of disease activity in patients with RA and UA (n = 397) with different combinations of ACPA, RF and anti-CarP. Association between CRP (A), ESR (B), SJC (C) and DAS28 (D) and different autoantibody combinations. Horizontal lines represent median. Whiskers show the 10th–90th percentile. Dots represent outliers. ACPA anti-citrullinated protein antibodies; RF: rheumatoid factor; anti-CarP: anti-carbamylated protein antibodies, CRP C-reactive protein, ESR erythrocyte sedimentation rate, SJC swollen joint count based on 66 joints, DAS28 disease activity score in 28 joints. *Significant difference between subgroups (p < 0.05). (JPG 104 kb) [file 13075_2016_1076_MOESM5_ESM.jpg]
